# Influence of Hepatitis C Virus and *IL28B* Genotypes on Liver Stiffness

**DOI:** 10.1371/journal.pone.0115882

**Published:** 2014-12-29

**Authors:** Lene Fogt Lundbo, Louise Nygaard Clausen, Nina Weis, Kristian Schønning, Lene Rosenørn, Thomas Benfield, Peer Brehm Christensen

**Affiliations:** 1 Department of Infectious Diseases, Hvidovre Hospital, University of Copenhagen, Copenhagen, Denmark; 2 Clinical Research Centre, Hvidovre Hospital, University of Copenhagen, Copenhagen, Denmark; 3 Department of Clinical Medicine, Faculty of Health and Medical Sciences, University of Copenhagen, Copenhagen, Denmark; 4 Department of Clinical Microbiology, Hvidovre Hospital, University of Copenhagen, Copenhagen, Denmark; 5 Department of Infectious Diseases, Odense University Hospital, Odense, Denmark; National Institute of Allergy and Infectious Diseases, United States of America

## Abstract

**Objective:**

Liver fibrosis has been associated with hepatitis C virus (HCV) genotype and genetic variation near the interleukin 28B (*IL28B*) gene, but the relative contribution is unknown. We aimed to investigate the relation between HCV genotypes, *IL28B* and development of liver stiffness.

**Patients and Methods:**

This cross-sectional study consists of 369 patients with chronic hepatitis C (CHC). Liver stiffness was evaluated using transient elastograhy (TE). Factors associated with development of liver fibrosis were identified by logistic regression analysis.

**Results:**

We identified 369 patients with CHC. 235 were male, 297 Caucasians, and 223 had been exposed to HCV through intravenous drug use. The overall median TE value was 7.4 kPa (interquartile range (IQR) 5.7–12.1). HCV replication was enhanced in patients carrying the *IL28B* CC genotype compared to TT and TC (5.8 vs. 5.4 log_10_ IU/mL, p = 0.03). Patients infected with HCV genotype 3 had significantly higher TE values (8.2 kPa; IQR, 5.9–14.5) compared to genotype 1 (6.9 kPa; IQR, 5.4–10.9) and 2 (6.7 kPa; IQR, 4.9–8.8) (p = 0.02). Within patients with genotype 3, *IL28B* CC genotype had the highest TE values (p = 0.04). However, in multivariate logistic regression, using various cut-off values for fibrosis and cirrhosis, only increasing age (odds ratio (OR) 1.09 (95% confidence interval (CI), 1.05–1.14 per year increment)), ALT (OR 1.01 (95% CI, 1.002–1.011), per unit increment) and HCV genotype 3 compared to genotype 1 (OR 2.40 (95% CI, 1.19–4.81), were consistently associated with cirrhosis (TE>17.1 kPa).

**Conclusions:**

Age, ALT and infection with HCV genotype 3 were associated with cirrhosis assessed by TE. However, *IL28B* genotype was not an independent predictor of fibrosis in our study.

## Introduction

It is estimated that about 170 million people are infected with hepatitis C virus (HCV) world-wide, and that more than 350.000 people die annually from chronic hepatitis C (CHC) related end-stage liver disease [1].

The influence of HCV genotypes in development of liver fibrosis is not well established. There are 6 epidemiologically important genotypes. In Denmark, genotypes 1 and 3 predominate [2]. Numerous subtypes (e.g., subtypes 1a and 1b within genotype 1 and subtypes 3a and 3b within genotype 3) exist. HCV genotype 1 has been associated with higher mortality, AIDS in HIV/HCV coinfected, and hepatocellular carcinoma [3]–[5], whereas HCV genotype 3 has been associated with development of hepatic steatosis, fibrosis and progression to cirrhosis in some, but not all, studies [6]–[10].

Several genome-wide association studies have revealed that the *IL28B* single-nucleotide polymorphism (SNP), rs12979860 genotype CC is advantageous in predicting the probability of spontaneous clearance and sustained virologic response following pegylated-interferon and ribavirin treatment in genotype 1 infected patients [11]–[13].

Recent publications of both HCV monoinfected and HIV-HCV coinfected patients, have studied the role of *IL28B* and the development of liver steatosis and fibrosis. Using transient elastography (TE), Barreiro et al. reported an association of the CC genotype to liver fibrosis in coinfected individuals whereas Marabita et al. did not show any association as assessed by liver biopsy in monoinfected individuals [14], [15]. Ydreborg et al. and Rembeck et al. found an association between the CC genotype and more pronounced liver histopathology in HCV genotype 3 infection [16], [17]. Noureddin et al. was unable to show a difference in the frequency of fibrosis progression between patients with *IL28B* CC and non-CC genotypes but the *IL28B* CC genotype was associated with hepatic necro-inflammation, higher alanine aminotransferase (ALT) levels, and worse clinical outcomes [18]. Similarly, two studies of steatosis came to conflicting results [19], [20].

We aimed to investigate the relation between HCV genotypes and *IL28B* and development of liver fibrosis, assessed by TE, in patients infected with HCV.

## Patients and Methods

### Study population

This observational, retrospective study consisted of two independent cohorts of patients with CHC, seen consequetively in Department of Infectious Diseases, Odense University Hospital (OUH) or Department of Infectious Diseases, Hvidovre Hospital (HH) with a TE performed between March 2007 and December 2012. Inclusion criteria were HCV antibody positivity and one positive serum HCV RNA followed by a valid TE. All patients had a positive HCV antibody measurement prior to TE. Exclusion criteria were HCV treatment within 6 months prior to TE, any other cause of liver disease, hepatitis B virus and HIV infection. Demographic, clinical and laboratory parameters were collected from medical records by a physician.

According to Danish Legislation, the Research Ethics Committee can grant an exemption from obtaining informed consent for research projects based on biological material under certain circumstances, and for this study such an exemption was granted.

The study was approved by the Danish Data Protection Agency (record no. 2012-231-0031).

### Laboratory

Participants were diagnosed with HCV infection on the basis of anti-HCV detected in a serum or plasma sample by 3^rd^ generation enzyme-linked immunosorbent assay and confirmed by recombinant immunoplot assay or HCV RNA [21], [22]. All samples were tested for RNA levels in the real-time polymerase chain reaction (PCR) system COBAS AmpliPrep/COBAS TaqMan HCV (CAP/CTM HCV) [23]; the detection limit was 15 IU/ml.

HCV genotype was determined with genotype specific primers from the 5' noncoding region of the virus by RT-PCR [24] or by sequence analysis of RT-PCR generated fragments using C/E1 and NS5B-specific primers [25], [26].

### *IL28B* genotyping

For patients from HH genotyping of the *IL28B* rs12979860 SNP (hereafter refered to as the *IL28B* SNP) were performed by LGC Genomics (Herts, United Lingdom) using a KASP competitive allele-specific PCR [27]. For a minority, genotypings were done by Molecular Genetics, Department of Clinical Biochemistry Department, Copenhagen University Hospital. Patients at OUH were tested by a SNP genotyping assay using real-time PCR(Lifetechnologies).

### Transient Elastography

TE was determined by Fibroscan (EchoSens, Paris, France) and was considered successfull if 10 valid measurements were obtained (valid measurements >60% and interquartile range <25%). TE was expressed in kilopascal (kPa) [28]. Cut-off values for liver stiffness and different degrees of fibrosis were assessed by two methods. Firstly, advanced liver fibrosis was defined as a TE value >9.5 kPa [29], [30]. Secondly, recent simplified cut-off values were used: no fibrosis: ≤7.0 kPa; fibrosis: 7.1–17.1 kPa; and cirrhosis: >17.1 kPa [31].

### Statistical analysis

Values are expressed as median, IQR and percent. Differences were compared using the Kruskal-Wallis test or the Wilcoxon Signed Rank Test for continuous variables and for categorical variables using X^2^ or Fisher's exact test, as appropriate. Logistic regression analyses using TE cut-off defined above were performed to evaluate predictors of fibrosis after adjusting for age, HCV genotype and *IL28B* genotypes and results were presented as OR with 95% CI. Genotype equilibrium was tested by the method of Hardy and Weinberg [32]. All statistical analyses were performed using Statistical Analysis Systems (SAS version 9.3; SAS Institute, Cary, NC, USA) or IBM SPSS Statistics (version 20; IBM, Armonk, New York, USA).

## Results

### Patient characteristics

The study included 369 patients; 230 patients from OUH and 139 from HH. The majority of these were male (64%; 95%CI, 59–69%), Caucasians (80%; 95% CI, 76–85%), and had been exposed to HCV through injection drug use (IDU) (60%; 95% CI, 55–65%). The median time of known HCV seropositivity was 26.3 (IQR, 3.2–96.9) months and the median age was 48 (IQR, 39–54) years at the time of TE. For all HH patients ALT was obtained within a 182 days time range relative to the fibroscan. For forty-three of the OUH patients ALT was not provided within 6 months prior to or after the fibroscan. The first obtained HCV RNA was available for all patients. HCV RNA within a 180 days range from the TE was available for 218 patients (135 HH patients and 83 OUH patients). No statistical significant difference was observed between the two RNA values (p = 0.09).

The majority of the patients were infected with HCV genotype 1 (50%; 95% CI, 45–55%), followed by genotype 3 (40%; 95% CI, 35–45%) and genotype 2 (9%; 95% CI, 7–13%). Only 8 patients were infected with genotype 4 and these patients were not considered further.

There was no difference in sex, route of exposure or known duration of infection according to HCV genotype, but patients infected with genotype 3 were younger (p = 0.001) than patients infected with genotype 1 or 2. Genotype 1 was more frequent among Caucasians (p = 0.03). The main characteristics of the study population are depicted in Table 1. The patients were either hepatitis C treatment naïve at the time of - or had relapsed at least 6 months prior to - the TE. Fourteen patients had been treated prior to their TE.

**Table 1:** Patient characteristics for 369 patients with chronic hepatitis C virus infection by HCV genotype.

	Genotype 1	Genotype 2	Genotype 3	Total	p-value
	n (%)	n (%)	n (%)	n	
	185 (50)	35 (10)	149 (40)	n = 369	
***Sex***					0.28^**^
Male	120 (65)	18 (51)	97 (65)	235 (64)	
Female	65 (35)	17 (49)	52 (35)	134 (36)	
**Age at TE, years**	49 (37–55)	54 (46–57)	46 (38–53)	48 (39–54)	0.001^***^
***Race***					0.03^**^
Caucasian	159 (86)	26 (74)	112 (75)	297 (80)	
Non-Caucasian/unknown	26 (14)	9 (26)	37 (25)	72 (20)	
***Route of exposure***					0.17^**^
Injection drug use	109 (59)	17 (49)	97 (65)	223 (60)	
Other/unknown	76 (41)	18 (51)	52 (35)	146 (40)	
**Length of known HCV infection, months**	18.8 (2.8–86.1)	45.6 (2.9–107.4)	36.1 (3.9–104.1)	26.3 (3.2–96.9)	0.27^***^
**Previously received antiviral treatment** ‡					0.50^**^
Yes	40 (22)	6 (17)	38 (26)	84 (23)	
No	145 (78)	29 (83)	111 (75)	285 (77)	
**HCV RNA, log_10_ IU/mL**	5.9 (5.1–6.5)	5.5 (4.7–5.7)	5.5 (4.7–5.8)	5.6 (4.9–6.2)	0.003^***^
**ALT, U/L**	63 (38–94)	47 (26–96)	71 (42–147)	64 (38–111)	0.01^***^
**Liver stiffness, kPa**	6.9 (5.4–10.9)	6.7 (4.9–8.8)	8.2 (5.9–14.5)	7.4 (5.7–12.1)	0.02^***^
***Liver stiffness stage***					0.15^**^
No fibrosis (≤7.1 kPa)	95 (51)	20 (57)	65 (44)	180 (49)	
Fibrosis (7.1–17.1 kPa)	66 (36)	12 (34)	52 (35)	130 (35)	
Cirrhosis (>17.1 kPa)	24 (13)	3 (9)	32 (25)	59 (16)	
**IL28B ** ***genotype*** ^†^					0.0005*
CC	48 (27)	19 (56)	62 (45)	129 (37)	
CT	106 (60)	10 (29)	67 (48)	183 (52)	
TT	23 (13)	5 (15)	10 (7)	38 (11)	

TE, transient elastography.

^*^Fisher's exact test, ^**^x^2^ or ^***^kruskall-wallis applied as appropriate.

† rs12979860.

‡ Relapsed more than 6 months prior to TE or treatment initiated after TE. All continous variabels reported as median and interquartile range

At HH, more patients were infected with HCV through IDU compared to OUH (69% vs 58% p = 0.03), and the patients from HH were older (50 years (IQR 42–55) vs. 46 years (IQR 36–53), p = 0.0008). There was no difference in sex, race, frequency of HCV genotypes and *IL28B* genotypes between the two cohorts.

### Viral load

Homozygous CC carriers had a significantly higher (first obtained) viral load compared to TC and TT carriers (median 5.8 and 5.4 log_10_ IU/mL, p = 0.03).

The HCV genotype 2 and 3 infected homozygous CC carriers had a higher (first obtained) median HCV RNA compared to CT and TT combined (median 5.6 vs. 5.1 log_10_ IU/mL for genotype 2 and 5.6 vs. 5.0 log_10_ IU/mL for genotype 3). This significant difference was not observed among the genotype 1 infected patients.

HCV genotype 1 infected patients had significantly higher viral load compared to genotype 2 and 3 infected at the time of TE (table 1).

### *IL28B* rs12979860

The majority of our study cohort (350 of 369 (95%)) was genotyped for the *IL28B* SNP. We lacked DNA to genotype the remaining 19 patients. The overall distribution of the alleles was as follows (no. of patients, %): CC (129, 37%), CT (183, 52%), TT (38, 11%). The allele distribution was in Hardy-Weinberg disequilibrium (p<0.05). *IL28B* genotype differed according to HCV genotype. Genotype CC was less frequent among genotype 1 infected patients compared to genotype 2 and 3, and consequently CT and TT (non-CC) were more frequent (P<0.005) (Table 1). Because prior studies have shown that *IL28B* allelic traits behave as an autosomal recessive inheritance [14] and due to the very few cases of TT, the CT and TT genotypes were combined in regression analyses (see below).

No association was found between ALT and *IL28B* genotypes.

### Liver stiffness

The overall TE value was 7.4 (IQR, 5.7–12.1) kPa. Patients infected with HCV genotype 3 had significantly higher TE values (8.2 kPa; IQR, 5.9–14.5) compared to genotype 1 (6.9 kPa; IQR, 5.4–10.9) and genotype 2 (6.7 kPa; IQR, 4.9–8.8) (p = 0.02).

Overall, 59 (16%) met the criteria of cirrhosis (TE>17.1 kPa), and 132 (36%) had fibrosis (Table 1).

For genotype 1 and 2, TE values did not differ according to *IL28B* genotype. For genotype 3, patients with the *IL28B* CC genotype had significantly higher TE values (10.0 kPa; 6.2–16.8) than patients with TC (7.6 kPa; 5.7–14.3) or TT (5.6 kPa; 4.7–7.7) (P = 0.04, Table 2).

**Table 2:** Association between HCV genotype, *IL28B* genotype and results of transient elastography for 369 patients with chronic hepatitis C virus infection.

	HCV genotype 1, (n = 185)				HCV genotype 2, (n = 35)				HCV genotype 3, (n = 149)			
*IL28B* genotype	CC (n = 48)	TC (n = 106)	TT (n = 23)	p-value	CC (n = 19)	TC (n = 10)	TT (n = 5)	p-value	CC (n = 62)	TC (n = 67)	TT (n = 10)	p-value
**Transient Elastography, kPa (IQR)**	6.9 (5.4–11.1)	7.4 (5.3–11.9)	8.1 (6.4–11.9)	0.61^***^	7.1 (5.8–10.0)	5.9 (4.7–6.3)	7.6 (7.5–7.8)	0.22^***^	10.0 (6.2–16.8)	7.6 (5.7–14.3)	5.6 (4.7–7.7)	0.04^***^
**Liver stiffness stage**				0.78^*^				0.12^*^				0.26^*^
No fibrosis (≤7.1 kPa)	25 (52)	52 (49)	10 (43)		10 (53)	8 (80)	1 (20)		23 (37)	32 (48)	7 (70)	
Fibrosis (7.1–17.1 kPa)	18 (38)	40 (38)	8 (35)		7 (37)	1 (10)	4 (80)		24 (39)	21 (31)	3 (30)	
Cirrhosis (>17.1 kPa)	5 (10)	14 (13)	(22)		2(10)	1 (10)	0 (0)		15 (24)	14 (21)	0 (0)	

*Fisher's exact test, ^**^x^2^ or ^***^kruskall-wallis applied as appropriate. All values are median and interquartile range.

### Factors associated with advanced fibrosis (TE>9.5 kPa)

By univariate logistic regression analysis, increasing age, ALT and HCV genotype 3 were each associated with advanced liver fibrosis (TE>9.5 kPa) whereas sex, race, route of exposure, duration of known seropositivity, viral load, *IL28B* genotypes or center were not. In multivariate analysis, increasing age (OR 1.08 (95% CI, 1.05–1.12) per year increment), ALT (OR 1.01 (95% CI,1.004–1.012) per unit increment) and HCV genotype 3 compared to genotype 1 (OR 1.91 (95% CI, 1.10–3.33) remained statistically significantly associated with advanced liver fibrosis. There was a trend towards less liver stiffness for genotype 2 compared to genotype 1 (OR 0.47 (95% CI, 0.17–1.30).

In genotype 3 patients, the *IL28B* CC genotype was associated with a two-fold increased risk of advanced liver fibrosis in the unadjusted analysis (OR 2.09, 95% CI 1.05–4.85)) compared to the CT/TT genotype. In multivariate analysis adjusted for age and ALT this association remained but lost statistical significance (OR 1.58, 95% CI 0.66–3.75).

For HCV genotype 1 (OR 0.78, 95% CI, 0.33–1.86) and genotype 2 (OR 2.13, 95% CI, 0.32–14.08) there was no association between *IL28B* genotype and advanced liver fibrosis.

### Factors associated with cirrhosis (TE>17.1 kPa)

By univariate logistic regression analysis, increasing age, ALT and HCV genotype 3 were each associated with cirrhosis (TE>17.1 kPa) whereas sex, race, route of exposure, duration of known seropositivity, *IL28B* genotypes or center were not (table 3). In multivariate analysis, increasing age, ALT and HCV genotype 3 compared to genotype 1 remained statistically significantly associated with cirrhosis (table 3).

**Table 3:** Predictors of liver cirrhosis (liver stiffness >17.1 kPa).

	Unadjusted OR (95% CI)	Adjusted OR (95% CI)	P value
**Age, per year increment**	1.08 (1.05–1.12)	1.09 (1.05–1.13)	<0.0001
**Sex**		-	
Male	1.0		
Female	0.81 (0.45–1.46)		
**Route of exposure**		-	
Other	1.0		
IDU	0.87 (0.50–1.53)		
**Race**		-	
Caucasian	1.0		
Other	0.60 (0.27–1.33)		
**Time with HCV, per year increment**	1.04 (0.98–1.09)	-	
**1^st^ HCV RNA, log_10_ IU/mL**	0.75 (0.55–1.04)	-	
**ALT**	1.01 (1.00–1.01)	1.01 (1.00–1.01)	0.002
**Center**		-	
Odense University Hospital	1.0		
Hvidovre Hospital	0.90 (0.50–1.61)		
**HCV genotype**			0.01
1	1.0	1.0	
2	0.63 (0.18–2.21)	0.47 (0.11–1.99)	
3	1.84 (1.03–3.28)	2.55 (1.23–5.31)	
***IL28B*** ** genotype**		-	
TT/CT	1.0		
CC	1.13 (0.63–2.03)		

After stratification for HCV genotype, there was no association between *IL28B* CC genotype and cirrhosis for any HCV genotype.

All analyses involving *IL28B* were repeated separately for Caucasians in order to account for race specific effects but did not alter the results (data not shown).

## Discussion

In this cross-sectional study of patients infected with HCV, increasing age and HCV genotype 3 were associated with increased liver stiffness, while the *IL28B* CC genotype was not. Steatosis associated with HCV genotype 3 infection is a known risk factor for fibrosis [33], and the presence of steatosis in HCV genotype 3 infection is associated with higher HCV RNA viral load compared to other genotypes [34]–[36]. Several studies suggest that HCV replication could be enhanced in patients carrying the *IL28B* C allele [11], [37], [38]. It has been hypothesised that patients with HCV genotype 1 infection have “metabolic steatosis”, and HCV genotype 3 infected have ‘‘viral steatosis’’ [34], [39]. The mechanisms underlying viral steatosis caused by HCV genotype 3 are not fully elucidated.

Overall steatosis is an important cofactor in accelerating the development of hepatic fibrosis and necroinflammatory activity in patients with CHC [35]. This corresponds well with our findings of an increased risk of fibrosis in patients infected with HCV genotype 3.

For genotype 3, the *IL28B* CC genotype was associated with a higher TE value in univariate analysis. This association lost significance in multivariate analysis when adjusted for ALT and age. For HCV infected patients with elevated ALT it should be noted that inflammation of the liver is considered a confounding variable in analysis of liver stiffness [40]. ALT was not associated with *IL28B* genetic variants.

Barreira et al. showed that the *IL28B* CC genotype was associated with cirrhosis using a TE cut-off of 14.5 kPa [14]. By liver biopsy, Rembeck et al. showed that the CC genotype in HCV genotype 3 mono-infected patients entailed more pronounced portal inflammation and steatosis [16]. Similarly Ydreborg et al. reported that the CC SNP was associated with a higher TE value in HCV genotype 3 infected patients [17].

These mentioned studies differ from our study in several aspects. In the study of Barreiro et al. 86% were infected through IDU compared to 60% in our population, whereas HCV was transmitted through IDU in fewer patients in the study of Ydreborg et al. [14], [17]. Due to sample size, we might lack statistical power in our study.

Other studies have assessed liver fibrosis by liver biopsy, whereas we used TE. TE is known to be a valid tool for estimating fibrosis but liver biopsy is still considered the gold standard [30]. A recent study by Degos et al. suggested that TE and liver biopsy performed comparable with regard to a diagnosis of cirrhosis whereas liver biopsy was superior in diagnosing fibrosis [31].

Various transient elastography cut-off values to define fibrosis and cirrhosis have been suggested [29]–[31]. We evaluated two of these. Using either of these, HCV genotype, ALT and age consistently were associated with fibrosis and cirrhosis, whereas the *IL28B* CC genotype only was not.

Further studies of a possible role of *IL28B* and other genetic variants in the development of fibrosis and cirrhosis are warranted.

Stengths of our study include the evaluation in two centers, and that we have a comparable number of HCV genotype 1 and 3 infected patients. Some limitations in our study must be acknowledged. First, TE is an indirect marker of liver fibrosis and several factors - most importantly elevated ALT - may overestimate fibrosis when determined by TE. Second, due to the retrospective design it was not possible to obtain information about certain potential confounding factors e.g. alcohol consumption among our patients. Third, it was not possible to estimate the exact duration of infection with HCV, which may explain the lack of an association between length of infection and cirrhosis. Most of our patients were diagnosed with HCV in their forties, but the vast majority has been infected years before detection. Genotype 3 infected patients had a median duration of known infection that was about twice as long as genotype 1 infected patients. However, this did not affect the results in the adjusted analysis. Fourth, the small numbers of carriers of the *IL28B* TT genotype infected with HCV genotype 3 restricted the comparison of the three *IL28B* genotypes separately.

In conclusion this two-center study evaluated the association of *IL28B* genotypes and the severity of liver fibrosis in HCV infected patients. We described a possible association of increased liver stiffness with HCV genotype 3 infection. Genetic variation in *IL28B* may influence fibrosis development in HCV genotype 3 infected individuals but further studies are required to confirm this.
